# Views of the L.C.C.

**Published:** 1920-01-10

**Authors:** 


### LONDON HEALTH SERVICES.

### Views of the L.C.C.

### THE NEED OF A LONDON HOSPITAL COUNCIL.

A SPECIAL committee of the London County Council has investigated, and now reports on, the question of the organisation of the health services of London, which are now administered by a multitude of authorities. The report frames proposals on a basis which will permit of a large measure of elasticity in such development, will not preclude important adjustments and alterations from time to time, and will meet the requirements of new legislation. In brief, the proposals provide for a consolidation of health services on a basis which will involve a minimum change in the existing organisation of London government. They assign to the central authority, the L.C.C., a measure of responsibility for uniformity and efficiency of local administration, besides providing sufficient flexibility to allow of modifications and adjustments to meet future needs as they arise.

A special recommendation is one for the formation of a Central Council of London Hospitals, and in this connection the report mentions that although a British Hospitals Committee exists, it does not appear to exercise any dominating influence on the hospital organisation as a whole.

The report deals exhaustively with present health services and indicates where they overlap.

Summarised, the leading recommendations are :—

(i.) The health functions of London Poor-Law authorities should be divided between the Council, the City Corporation, and the Metropolitan Borough Councils substantially on the basis proposed in the report of the Local Government Committee appointed by the Minister of Reconstruction on the subject of the transfer of functions of Poor-Law authorities in England and Wales.

(ii.) The administration in London of such additional health services as may by future legislation be required to be administered by public authorities should be entrusted to the Council, the City Corporation, and the Metropolitan Borough Councils, in addition to the duties already placed upon them by statute.

(iii.) The Council should be the central organising authority for the services referred to under (i.) and (ii.), and should be responsible for preparing such surveys of health services in London as may from time to time be required.

(iv.) Any exercise of powers by the Council as central organising authority should be governed by scheme to be prepared by the Council after consultation with the City Corporation and the Metropolitan Borough Councils, and submitted by the Council to the Minister of Health for approval.

(v.) The Council should have power, by scheme, to appoint a health committee, of whom a majority should be members of the Council and a minority persons of experience in health matters who are not members of the Council, and to refer to such Committee such duties as are set out in the scheme, including (should the Council hereafter consider such inclusion desirable) any or all duties regarding the medical inspection and treatment of school children at present standing referred by statute to the Education Committee.

(vi.) The cost of the public-health services of environmental or entirely local character should be borne by local funds.

(vii.) The cost of prevention of the spread of infectious diseases should be borne as to not less than one half by national funds and as to the balance by local funds.

(viii.) In the case of the medical treatment of school children a charge should continue to be made by the authority responsible for the provision of such treatment.

(ix.) In connection with medical treatment provided by public authorities in cases other than those under (vi.) to (viii.) inclusive above, the general principle should be recognised that the cost (excluding expenses of administration) of such treatment should be borne, to the extent of his ability to pay, by the person treated or by a fund, such as the insurance fund, made up mainly by contributions of employers and employed.

In regard to the voluntary health agencies, the report refers to the great voluntary hospitals which have made an important contribution to the provision existing for the needs of the sick of London. The vastness of their work is recognised in the following paragraph :

"Practically the whole of the institutional provision, other than that made by the Poor-Law authorities, for the treatment of non-infectious diseases has been made by the voluntary hospitals of London, which, it may be mentioned incidentally, draw their patients from a far wider area than that of the administrative county. The assumption by the public authorities of the responsibility for preventing the spread of infectious diseases has tended to prevent any considerable extension in recent years by the voluntary hospitals of the provision for these diseases. Such fresh provision as has been made by the voluntary hospitals has been inspired more from the point of view of the treatment and cure of the individual than from a desire to segregate him and thus prevent the spread of the disease to others."

The report declares that any scheme covering the whole field of health administration in London would pay the fullest regard to the position of the voluntary hospitals, which have a long record of valuable work for the benefit of the people of London. It is thought that it would be of considerable advantage if, in dealing with questions affecting the whole of London, the Council were able to deal with a central body representing the London hospitals, on which official bodies, such as the Ministry of Health and the Council, could be represented. Such a proposal is not a new one. So long ago as 1892 a Select Committee of the House of Lords recommended the establishment of a central body representing the hospitals and dispensaries of the Metropolis and some other institutions specially interested in medical relief, and indicated the functions which such a central body should perform for purposes of co-operation, co-ordination, and improvement in methods of administration. No such central body has yet been formed, but the governing body of King Edward's Hospital Fund for London has undertaken during the past twenty years some of the functions indicated by the Select Committee as desirable for a central body. A representative Council for the London hospitals might thus be similar in constitution to the Central Council for District Nursing in London, but with a larger proportionate voluntary representation. Such a Hospital Council would have a recognised position in the general scheme for health administration in London.

